# Why usefulness is rarely useful

**DOI:** 10.1093/g3journal/jkae296

**Published:** 2024-12-24

**Authors:** Fangyi Wang, Mitchell J Feldmann, Daniel E Runcie

**Affiliations:** Department of Plant Sciences, University of California Davis, Davis, CA 95616, USA; Department of Plant Sciences, University of California Davis, Davis, CA 95616, USA; Department of Plant Sciences, University of California Davis, Davis, CA 95616, USA

**Keywords:** phasing errors, family variance, usefulness criterion, cross selection, Genomic Prediction, GenPred, Shared Data Resource

## Abstract

Mate selection plays an important role in breeding programs. The Usefulness Criterion was proposed to improve mate selection, combining information on both the mean and standard deviation of the potential offspring of a cross, particularly in clonally propagated species where large family sizes are possible. Predicting the mean value of a cross is generally easier than predicting the standard deviation, especially in outbred species when the linkage of alleles is unknown and phasing is required. In this study, we developed a method for estimating phasing accuracy from unphased genotype data on possible parental lines and evaluated whether the accuracy was sufficient to predict family standard deviations of possible crosses. We used simulations spanning a wide range of genetic architectures and used genotypes from a real strawberry breeding population to evaluate the conditions when usefulness could be accurately predicted. We found that with highly accurate computational phasing, predicting family standard deviations and usefulness criteria for potential crosses yields benefit over simply selecting crosses based on predicted family means only at high selection intensity and high heritability and with small numbers of QTL. However, even then the gain from using the family usefulness is small.

## Introduction

Plant breeding programs aim to create genotypes with improved performance in one or more target traits. New genotypes are created by making crosses between existing genotypes, using recombination and Mendelian assortment to assemble sets of alleles that did not exist. These new genotypes can then be evaluated and released as commercial products or recycled to create new crosses. The success of a breeding program can be improved by evaluating genotypes more efficiently and making crosses that are more likely to generate better genotypes (Meuwissen *et al.* 2001; Heffner *et al.* 2009; Bernardo 2014; Forster *et al.* 2015). In recent years, genomic data have been leveraged in many breeding programs to make both steps more effective. Genomic data can help make the evaluation of genotypes based on phenotypic data more precise by modeling the correlations between phenotypes and genotypes. Genomic data can also be used to select parents to be crossed by predicting their breeding value (BV), or the expected average performance of their offspring (Meuwissen *et al.* 2001; Heffner *et al.* 2009; Jannink *et al.* 2010). Both uses of genomic data are now commonly implemented in plant breeding programs.

Predicting the breeding value of potential parents is widely used to choose crossing partners. When the number of offspring per cross is large, the best crosses are the ones that will create the best possible offspring, not the crosses that will make the best average offspring. This situation is common in many plant systems because large numbers of seeds can be generated from the same cross. This result was observed nearly 50 years ago by Schnell and Utz (1975), who proposed the usefulness criterion (UC), or usefulness, to design optimal crosses (Zhong and Jannink 2007). Usefulness is defined as


$${\mathrm{UC}}_{j}=\mu_{j}+i\sigma_{j},$$

where $\mu_{j}$ is the average genetic value of offspring of cross *j*, $\sigma_{j}$ is the standard deviation (SD) of genetic values among these offspring, and *i* is within family selection intensity, the difference in mean phenotypic value of selected offspring from each family relative to the family’s mean. The consequence of the usefulness is that crosses between parents that individually have good breeding values are not necessarily the best to make. For example, the upper tail of the distribution of genetic values of offspring from a cross with high variance (high $\sigma_{j}^{2}$) may have higher values than the upper tail of the distribution of offspring from a family with higher mean but lower variance. Selection decisions based on usefulness have received little attention in animal breeding, possibly because the number of offspring per cross is very small so the chance of creating offspring in the tails is low. However, several recent plant breeding studies have suggested that usefulness-based breeding might be successful, particularly in clonally propagated crops (Wolfe *et al.* 2021).

A simple method to predict the mean breeding value of a family is to use the average of the breeding value of the two parents. This will be accurate as long as most QTL operate additively. However, even under additive gene actions, predicting the variance within a family is more challenging as patterns of co-segregation of causal loci among genotypes also contribute to variance. For example, a cross between two genotypes with two coupling heterozygous loci would produce a greater variance in the next generation, compared with one with two repulsion heterozygous loci (Lynch and Walsh 1998; Zhong and Jannink 2007; Wolfe *et al.* 2021). Predicting co-segregation of causal loci from a cross of two outbred parents requires information on the phasing of alleles between all pairs of heterozygous loci in each parent and phased haplotypes are not directly measured by most genotyping platforms when individuals are heterozygous. Computational phasing using software like Beagle (Browning and Browning 2007; Browning *et al.* 2018) and FILLIN (Swarts *et al.* 2014) have been used in plant populations (Poland and Rife 2012; Scott *et al.* 2020). However, whether these methods are sufficiently accurate to predict family variance is unknown. Once phased haplotypes are known, QTL effect sizes need to be estimated in a training population, and then either family population variance (Zhong and Jannink 2007; Osthushenrich *et al.* 2017) or sample variance can be predicted (Zhong and Jannink 2007; Lehermeier *et al.* 2017; Osthushenrich *et al.* 2017; Wolfe *et al.* 2021; Bernardo 2022).

The benefit of choosing crosses based on their usefulness will likely vary among crops and breeding programs due to differences in genetic architecture, breeding system, and training population size. Zhong and Jannink (2007) evaluated the benefit of the usefulness in inbred populations by analytical studies and simulations. They found a generally limited utility of usefulness in inbred populations. A recent study by Wolfe *et al.* (2021) evaluated the usefulness in a cassava breeding program with pre-phased parental individuals and empirical observations of cross-variance in 4 traits. They argued that both family mean and variance were important metrics for mate selection. In their results, the family mean and usefulness criterion were correlated and similar selections were made using either criterion. This was one of the first applications of usefulness in an outbred, clonally propagated species using whole-genome marker data and genomic prediction models of marker effects. Our work focuses on strawberry, another outbred, clonally propagated species, and on the University of California Davis (UCD) strawberry breeding program. This breeding program is currently investing in infrastructure to implement genomics data in breeding decisions (Pincot *et al.* 2022; Hardigan *et al.* 2023; Jiménez *et al.* 2023; Feldmann *et al.* 2024b; Knapp *et al.* 2024) and has detailed records of the pedigrees of current varieties (Pincot *et al.* 2021; Knapp *et al.* 2023; Feldmann *et al.* 2024a) which help evaluate the success of the statistical models necessary for predicting usefulness as detailed below.

Our study builds on the work of Zhong and Jannink (2007) and Wolfe *et al.* (2021) in three key ways. First, we develop and apply a method to measure the accuracy of computationally phased parental haplotypes which is necessary for accurately predicting usefulness. Second, our study uses a different reference population with a different pedigree topology and a different marker density. Zhong and Jannink (2007) studied the benefit of usefulness in inbred species, while our study focuses on an outcross species. Therefore, our results test whether their conclusions are robust in other systems. Finally, we use numerical simulations of genetic architectures so that we can compare computationally predicted usefulness values to the actual (simulated) values without measurement error. We repeat these simulations across a range of additive genetic architectures from oligogenic to polygenic and from low to high heritability so that our conclusions can generalize to many possible target traits. Overall, our results show that despite highly accurate computational phasing, the conditions under which selection decisions based on usefulness could be useful are relatively rare. This is consistent with earlier results from Bernardo (2022) who studied predicting genetic variance within biparental populations created from inbred parents and concluded that there was little benefit to modeling within family genetic variance.

## Methods and materials

### Genetic data and genotyping error estimation

We used genetic and pedigree data from a population of 1,007 strawberry genotypes with 37,441 SNPs. We identified 593 family trios based on the pedigree to assess genotyping error rates and pedigree accuracy. Each trio is composed of two parents and one of their progeny. These 593 progenies were not parents of any other genotypes in the pedigree. The parents of the 593 progenies were included in the rest of the 414 genotypes. Within each family trio, we examined SNP markers for which both parents were homozygous. We compared the parental and progeny genotypes at those markers and counted the number of mismatches between parents and progeny. For example, parental genotype *AA* and *aa* with a progeny genotype *AA* were considered a mismatch. The genotyping error was calculated as the number of mismatched markers divided by the total number of homozygous markers compared per trio. All of the family trios had a genotyping error rate of <5%.

### Phasing

To predict the family breeding value variance, the linkage between heterozygous alleles within each parent must be known. Assigning heterozygous genotype calls to haplotypes is phasing. We used Beagle 5.0 (Browning and Browning 2007; Browning *et al.* 2018), version *05May22.33a* and seed 0, to phase the SNP data for all 1,007 genotypes and to impute any missing genotype calls. We used a genetic map for the markers with 28 pairs of chromosomes averaging 223 centiMorgans (cM), measured as the average distance between the furthest markers on the chromosomes. We excluded 2,530 of the 37,411 markers due to their unknown map positions or low allele frequencies.

### Simulation of offspring

We calculated family mean and standard deviation empirically by simulating crosses. We randomly selected 10,000 unique pairs of strawberry genotypes as parents for the crossing simulation. For each pair of parents, we used the *Hypred* R package (Technow 2011) to simulate crossover events and produce 400 gametes using the same genetic map as above, and the gametes were randomly paired to produce 200 offspring. Crossover events were simulated based on a count-location model. The number of crossover events (on a single chromosome) followed a Poisson distribution Pois(L), where *L* was the length of the chromosome in Morgans. The locations of crossover events were sampled from a uniform distribution U(0,L).

### Estimation of imputation and phasing error

To measure the accuracy of phasing and imputation, we re-ran Beagle using the 414 genotypes that were not the progenies of the 593 family trios, and left the 593 progenies unphased. We estimated the imputation error using the same method of estimating the genotyping error as above. We compared genotypes of progeny to their computationally phased parents at pairs of markers for which at least one parent was heterozygous at both markers (e.g. *AaBb* and *AABB* parents). We measured the percentage of progeny genotypes that were inconsistent with the predicted parental haplotypes for markers, binned by the cM distance between markers.

We compared the calculated rates of phasing inconsistencies to the expected rates of inconsistencies given our genetic map, by introducing phasing errors in the haplotypes of each parent and then comparing the “incorrect” parent haplotypes to the simulated offspring genotypes using the “correct” parent haplotypes. We estimated the rate of offspring genotype inconsistency with the “incorrect” parental haplotypes using the method above. We defined 6 rates of phasing error per cM distance p∈(0.005,0.01,0.025,0.05,0.1,0.15) as the probability of a phasing error between two SNPs 1 cM apart. Phasing errors were introduced using the same method of crossover events as described above. The number of phasing errors per chromosome was sampled from a Poisson distribution Pois(p*L), where *L* is the chromosome length in Morgans. The locations of phasing errors were sampled from a uniform distribution U(0,L).

### Simulation of breeding value and phenotypes

We simulated breeding values assuming a sparse additive genetic architecture. We created 15 scenarios spanning all combinations of three levels of heritability $h^{2}$ (0.2, 0.5, 0.8) and five levels of QTL number (4, 16, 64, 256, 1,024) randomly selected from the SNP markers with effect sizes sampled from a standard normal distribution N(0,1). We defined the BV of genotype *k* as $BV_{k}=\sum_{j}Z_{k,j}\alpha_{j}$, where $Z_{k,j}$ was the number of copies of alternative allele for genotype *k* at QTL *j* and $\alpha_{j}$ was the effect size of alternative allele at QTL *j*. To calibrate these architectures, we calculated the number of QTL segregating per pair of haplotypes, which is the average number of heterozygous QTL per individual. For the traits with 4 QTL, on average there were ≈1.5 segregating QTL per genotype, while for the traits with 1,024 QTL, there were ≈400 segregating QTL (Supplementary Fig. 3). Compared to a biparental population, this is equivalent to having 1.5 or 400 segregating loci. Focusing only on large-effect QTL explaining at least 20% of the variance in breeding values, haplotype pairs differed by ≈1.13 large-effect QTL when there were 4 QTL in the whole population, ≈3.43 when there were 16 QTL, and ≈ 7.58, 5.57, and 0.06 with 64–1,024 QTL (Supplementary Fig. 4).

We also simulated phenotypic values (*Y*) by adding random noise $\epsilon_{k}$ sampled from a normal distribution $N(0,\sigma_{\epsilon }^{2})$, i.e. $Y_{k}=BV_{k}+\epsilon_{k}$, where $\sigma_{\epsilon }^{2}$ was scaled based on different levels of heritability $h^{2}=\mathrm{Var}(BV)/(\mathrm{Var}(BV)+\sigma_{\epsilon }^{2})$. For each combination of heritability and QTL number, we ran 20 independent simulations, sampling different SNPs as QTL and with different effect sizes and environmental errors in each simulation, and we used 500 out of the 10,000 families for each simulation to ensure independence among the 20 simulations. We calculated breeding values for both the real 1,007 parental genotypes and simulated offspring, but the phenotypes were only simulated for the real 1,007 genotypes.

### Prediction of family means and standard deviation of breeding value, and estimation of prediction accuracy

RR-BLUP and BayesC models were fitted to the simulated phenotypes of the 1,007 parental genotypes and their SNP marker data, excluding the markers that were selected as QTL (Endelman 2011; Pérez and de los Campos 2014; Team 2020), i.e. $Y=X\;\hat{\;\beta }$, where Y and X were the phenotype vector and marker matrix of 1,007 genotypes and $\hat{\;\beta }$ was the trained models. Predicted breeding values ${\hat{BV}}_{\mathrm{offspring}}$ were calculated for each offspring by plugging offspring genotypes $X_{\mathrm{offspring}}$ into the fitted models, i.e. ${\hat{BV}}_{\mathrm{offspring}}=X_{\mathrm{offspring}}\;\hat{\;\beta }$. We calculated the within family breeding value mean and standard deviation based on both the true and predicted breeding value of offspring. For example, the true and predicted breeding value mean (*μ* and $\hat{\mu }$) of a family was calculated as $\mu =(\sum_{k}{BV}_{k})/200$ and $\hat{\mu }=(\sum_{k}{\hat{BV}}_{k})/200$, where *k* represented the *k*th offspring in the family. We calculated the prediction accuracy *r* as the Pearson correlation between the predicted values and the true values of each statistic (e.g. $r=\mathrm{cor}(\hat{\mu },\mu )$).

### Prediction of family mean and standard deviations of all possible crosses using simulation of gamates

We used the randomly simulated 10,000 families to assess the prediction accuracies for family mean, standard deviation, and usefulness. For mate selection, however, 10,000 crosses were only a small fraction of all 506,521 possible unique crosses that can be made among the 1,007 individuals, and the cross that yielded the highest mean or usefulness was likely not among them. We needed a method to exhaustively predict family means and standard deviations of all possible crosses without the simulation of offspring. For this method, we simulated 200 gametes from each of the 1,007 individuals using *Hypred* with the same count-location model.

We calculated the predicted breeding values of the gametes of each potential parent as ${\hat{BV}}_{\mathrm{gametes}}=X_{\mathrm{gametes}}\;\hat{\;\beta }$, where $\hat{\;\beta }$ was the trained BayesC model. We also calculated the predicted mean and variance of gametes from each parent, $\hat{\mu }$ and $\hat{\sigma }$. Since we only considered additive genetic architectures, we calculated predicted family means and variances of a cross as the summation of predicted means and variances of gametes from two individuals. For example, if a cross was made between individual *j* and individual *l*, the predicted family mean and variance would be calculated as ${\hat{\mu }}_{\;j,l}={\hat{\mu }}_{j}+{\hat{\mu }}_{l}$ and ${\hat{\sigma }}_{\;j,l}^{2}={\hat{\sigma }}_{j}^{2}+{\hat{\sigma }}_{l}^{2}$. Predicted family standard deviation was calculated as the square root of predicted family variance. We used this method to calculate the true and predicted family mean and standard deviations for all 506,521 crosses, using the simulated gametes. The estimates of family mean breeding values were highly correlated using both methods (>99%, Supplementary Fig. 7a). The correlations of standard deviations of breeding values within families were also high, but decreased as the number of QTL increased (from ∼99% to ∼75%, Supplementary Fig. 7b).

### Calculation of selection intensity

The usefulness criterion is a function of the standardized selection differential or selection intensity *i* defined in Caballero (2020). To compare true and predicted usefulness values, we selected two values of *i* representing moderate and strong selection: 1.40 and 2.67, corresponding to selecting 20 or 1% of offspring within each family, using the equation:


$$i=f\left(\frac{{\bar{Y}}_{\mathrm{selected}}-{\bar{Y}}_{\mathrm{population}}}{sd_{\mathrm{population}}}\right)/p.$$

Here, f(⋅) is the probability density function of the phenotypic distribution of the population, and we assume a normal distribution, and $({\bar{Y}}_{\mathrm{selected}}-{\bar{Y}}_{\mathrm{population}})/sd_{\mathrm{population}}$ represents how many population standard deviations the selected group mean is away from the population mean. *p* is the proportion of genotypes selected.

## Results and discussion

### Computational phasing is highly accurate in this strawberry population.

**Fig. 1.**
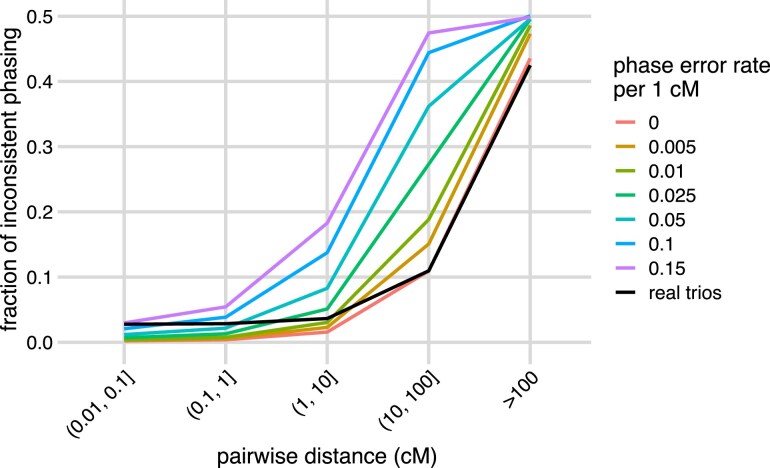
Rates of inconsistent parent–offspring genotypes at pairs of markers as a function of genetic distance. Pairs of marker genotypes, which both were heterozygous in at least one parent, were compared between parents and offspring across the 593 family trios. The black line shows the fraction of marker pairs in each genetic distance bin that are inconsistent between offspring and parents. Colored lines show simulated inconsistency rates for the same marker pairs after introducing haplotype switches (phasing errors) at the specified rates per cM in each parent.

We compared genotypes of pairs of markers at different genetic distances between unphased offspring and phased parents. To account for recombination, we binned marker pairs by genetic distances in cM. Rates of inconsistencies increased with recombination distance (Fig. 1), as expected. We then compared the observed rates of genotype inconsistency with expected rates if parents were correctly phased, or if phasing errors were introduced randomly within each parent with different frequencies. For marker pairs >10 cM apart, our observed rate of genotype inconsistencies was close to the expected rate if parental phasing was perfect (Fig. 1, black and red). However, for closer marker pairs, simulated inconsistency rates converged towards zero even when parental phasing was imperfect, but our observed inconsistency rate never dropped below ≈2.8%, perhaps due to a low amount of genotyping error or incorrect parent–offspring trios. These results are consistent with the computationally inferred phasing of markers being highly accurate in this population.

### Prediction accuracy of family breeding value mean and standard deviation varied as heritability and number of QTL changed

Given the highly accurate computational phasing of parental haplotypes in this population (Fig. 1), we used the haplotypes to simulate families of 200 offspring for 10,000 crosses between randomly paired genotypes and projected genotypes of causal alleles onto each simulated offspring. We then trained genomic prediction models (BayesC and RR-BLUP) on simulated parental phenotypes with varying levels of heritability ($h^{2}$) and used the trained models to predict the breeding values of the offspring (Endelman 2011; Pérez and de los Campos 2014). We calculated the mean and standard deviation of these predicted breeding values per family and measured the accuracies (*r*) of these predictions using Pearson’s correlation with the true values ($r=\mathrm{cor}(\hat{\mu },\mu )$, and $r=\mathrm{cor}(\hat{\sigma },\sigma )$).

**Fig. 2.**
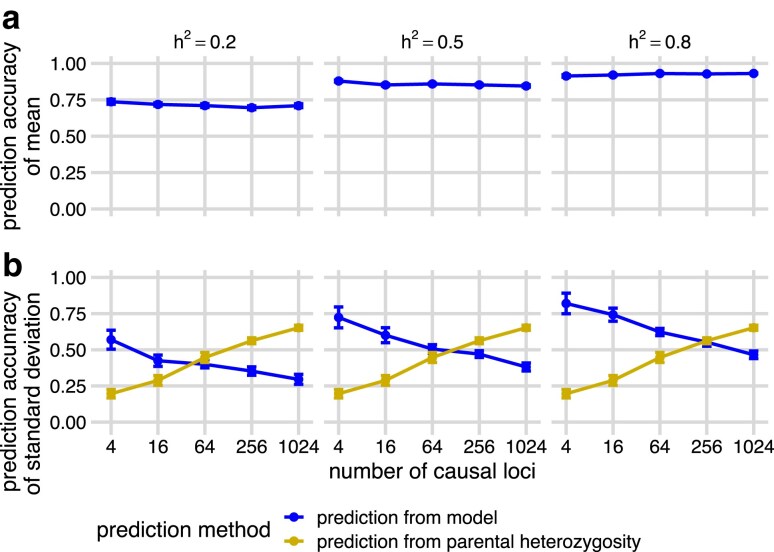
Prediction accuracies of the means a) and standard deviations b) of breeding values across simulated families as a function of heritability ($h^{2}$) and the number of causal loci. Panels show the means and standard error bars of Pearson’s correlations (prediction accuracies, *y*-axis) between the true family means of breeding values a) or true family standard deviations of breeding values (b, blue “prediction from model”) based on a BayesC genomic prediction model trained on parental phenotypes simulated with different heritabilities ($h^{2}$) and different number of causal loci (*x*-axis). Twenty simulations were run at each combination of $h^{2}$ and number of causal loci. Each simulation utilized 500 out of 10,000 simulated families to ensure independence among simulations. As a comparison, we also calculated the correlation between the true standard deviation of breeding values in each family and the average number of heterozygous markers of the two parents of that family calculated across all non-causal SNP markers in each simulation (b, gold “prediction from parental heterozygosity”). Points and bars show the means and standard errors of accuracies across 20 simulations. Curves are created by connecting the points of means and show the trend of prediction accuracies across simulations.

Using BayesC as a genomic prediction model, the average prediction accuracy of family means ranged between r=0.7 and r=0.9 (Fig. 2a). We achieved moderate accuracy in predicting family standard deviations, averaging between r=0.3 and r=0.8 across scenarios (Fig. 2b, blue “prediction from model”). Results using RR-BLUP as a prediction model were similar (Supplementary Fig. 1). The prediction accuracies of family breeding values mean and standard deviations both increased as the heritability increased, as expected. When the number of QTL increased, the prediction accuracy of the mean remained the same (Fig. 2a), but that of standard deviation decreased (Fig. 2b, blue “prediction from model”). This might be due to less accurate estimates of individual QTL effects when there are more QTL.

Even with a low heritability and a high number of causal loci, prediction accuracies of within family standard deviations did not decline to zero. This is likely because parents heterozygous for more loci also tend to be heterozygous for more causal loci. Only heterozygous causal loci contribute to the variance of breeding values among offspring in our simulations because the genetic architecture is purely additive. We used the number of heterozygous markers as a measure of an individual’s heterozygosity, and calculated Pearson’s correlation between the average parental heterozygosity and the true family standard deviation. The average parental heterozygosity was more correlated with the standard deviation of offspring breeding values than either the BayesC-based or RR-BLUP-based predicted standard deviations in some of our simulations (Fig. 2b, gold “prediction from parental heterozygosity”; Supplementary Fig. 1b, gold “prediction from parental heterozygosity”), particularly when the number of causal loci was high.

Mean heterozygosity information may have been indirectly captured by the genomic prediction models to accurately predict standard deviation. With a small number of QTL, the predicted family standard deviations from our genomic prediction models were more accurate than predictions of standard deviation from parental heterozygosity. In contrast, with a large number of QTL, the prediction of standard deviation from parental heterozygosity was more accurate. Unfortunately, despite its high correlation with family standard deviations in some instances, it is not straightforward to use parental heterozygosity to directly predict family usefulness, because heterozygosity and family standard deviations have different units and scaling factors. In practice, real families with large enough numbers of genotypes would be needed to estimate the regressions between family standard deviations and parental heterozygosity before this statistic could be used to estimate usefulness.

### Family usefulness can be accurately predicted with family mean

**Fig. 3.**
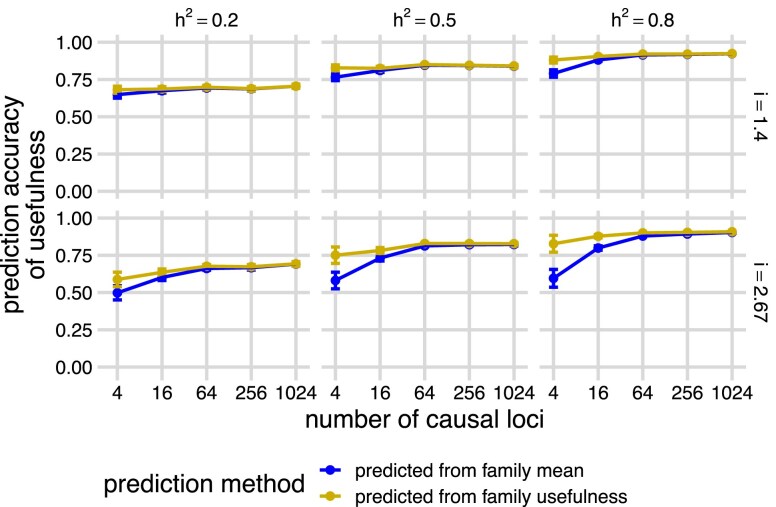
Prediction accuracies of the usefulness across the simulated families using a BayesC genomic prediction model. Panels show the means and standard error bars of Pearson’s correlations (prediction accuracies, *y*-axis) between the true family usefulness and predicted family mean (blue “predicted from family mean”) or predicted family usefulness (gold “predicted from family usefulness”) based on a BayesC genomic prediction model trained on parental phenotypes simulated with different heritabilities ($h^{2}$) and different numbers of causal loci (*x*-axis), across different selection intensity (*i*). Twenty simulations were run at each combination of $h^{2}$ and number of causal loci. Points and bars show the means and standard errors of accuracies across 20 simulations across each *i*. Curves are created by connecting the points of means and show the trend of prediction accuracies across simulations.

With the predicted family mean and standard deviation using the BayesC model, we were able to predict the usefulness of each family for a defined selection intensity *i* using the equation: $\hat{UC}=\hat{\mu }+i\hat{\sigma }$. We calculated the accuracy of the estimated usefulness as Pearson’s correlation with the true usefulness ($\mathrm{cor}(\hat{UC},UC)$, Fig. 3, gold “predicted from family usefulness”) and compared this with the accuracy of predicting the usefulness using only the predicted family mean ($\mathrm{cor}(\hat{\mu },UC)$, Fig. 3, blue “predicted from family mean”) of each family. As heritability increased, the accuracy of both predictions increased. At low heritability, the prediction accuracies were relatively insensitive to the number of QTL. However, at high heritability, the prediction accuracies increased as the number of QTL increased. Predicting usefulness with both predicted mean and predicted standard deviation (i.e. $\hat{UC}=\hat{\mu }+i\hat{\sigma }$) only achieved meaningfully higher accuracies than predicting usefulness with the predicted mean itself (i.e. $\hat{\mu }$), when heritability was high and when there were small numbers of QTL (Fig. 3, panels $h^{2}=0.5$ and $h^{2}=0.8$, at 4 and 16 QTL), the same conditions under which we could most accurately predict the family standard deviation (Fig. 2b, blue “predicted from family mean”). The results using an RR-BLUP model were similar (Supplementary Fig. 2).

**Fig. 4.**
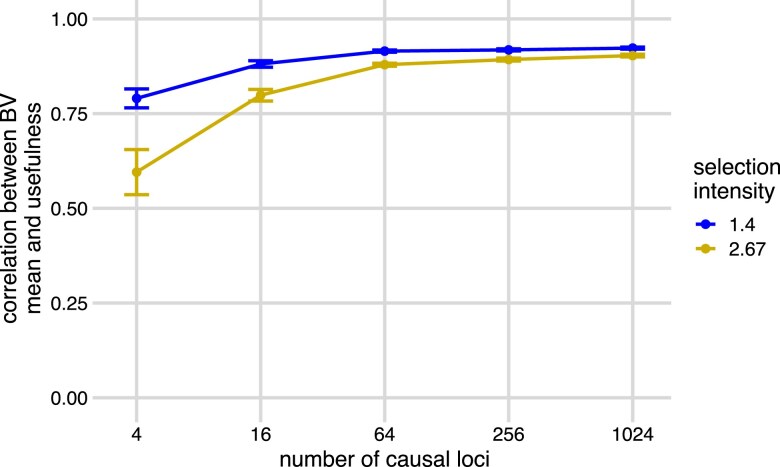
Correlations between true family breeding usefulness and mean at two selection intensity levels. Pearson’s correlation (*y*-axis) between the true family means of breeding values and true family usefulness across different selection intensities (*i*) and the number of causal loci (*x*-axis). Twenty simulations were run at each number of causal loci. Points and bars show the means and standard errors of accuracies across 20 simulations at i=1.4, blue, and at i=2.67, gold. Curves are created by connecting the points of means and show the trend of correlations across simulations.

Given the moderate accuracy at predicting both the standard deviation and mean of each family, we were surprised that direct predictions of usefulness were not much better than predictions based on predicted family means alone. One explanation for this observation is that the true values of family mean and family usefulness are highly correlated (cor(μ,UC)), especially when the number of causal loci is large (Fig. 4). The reason for this high correlation is that the variance among families in the quantity iσ (selection intensity multiplied by standard deviation) is very small compared with the variance among families in mean (*μ*), so the first term in the usefulness dominates. This mirrors observations from Zhong and Jannink (2007) in biparental recombinant inbred line populations that the variance of family means increases at a greater rate than the variance of family standard deviations as the number of QTL increases. They defined the parameter t=var(σ)/var(μ), the ratio between the variance of true family standard deviations and variance of means and showed that in biparental populations *t* accurately predicts the value of usefulness. We estimated *t* for each simulation (Supplementary Fig. 5, blue “simulated offspring”) and found that it also decreased dramatically as the number of causal loci increased. However, *t* was generally larger in our populations than expected based on Equation 8 of Zhong and Jannink (2007): $t=(4L{)}^{-1}$, where *L* is the effective number of causal loci (Supplementary Fig. 5, gold “the number of casual loci”), which assumes that all loci are independent, have equal allele frequencies (q=0.5), and equal effect sizes. The increase of *t* in our simulations is expected because there was LD among loci, unequal effect sizes, and unequal allele frequencies. This reduces the “effective” number of loci, as in Equation 9 of Zhong and Jannink (2007). However, that equation does not account for unequal allele frequencies, which increases *t* even more, as they predicted. Nevertheless, *t* values are still small in our simulations when there were four causal loci, which explains the negligible value of usefulness.

### Predicting usefulness improves cross-selection accuracy in a two-stage approach, but results in little additional genetic gain

**Fig. 5.**
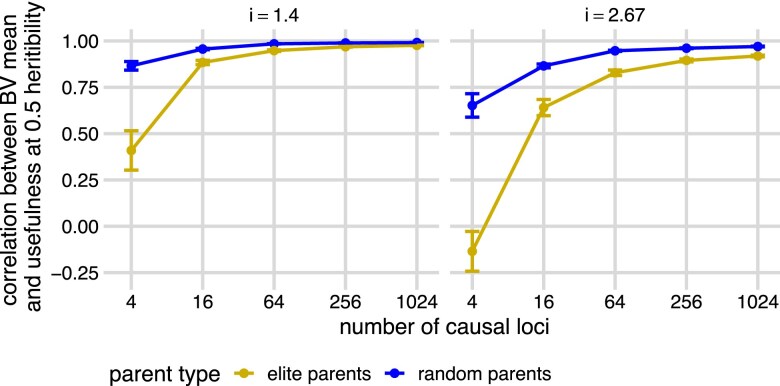
True family breeding value means and true family usefulness are less correlated in the elite families. Pearson’s correlation (*y*-axis) between the true family means of breeding values and true family usefulness across different selection intensities (*i*) and the number of QTL (*x*-axis), from the initial 10,000 families (blue “elite parents”) and from the families with the highest predicted breeding value individuals as parents (gold “random parents”) at 0.5 heritibility. Points and bars show the means and standard errors of accuracies across 20 simulations at i=1.4 and at i=2.67. Curves are created by connecting the points of means and show the trend of correlations across simulations.

Zhong and Jannink (2007) observed that by restricting possible crosses to only those among lines first selected for breeding value, (i.e. elites), the variance in family means will be reduced, and thus the relative importance of standard deviation for usefulness will increase. We tested whether our prediction accuracy of usefulness in this context could improve crossing decisions. Across the range of genetic architectures, the true correlation between the family mean breeding value and usefulness was lower among crosses from the elite parents than among random crosses across all numbers of causal loci and selection intensities for these new families, reaching values as low as 0 when selection intensities were strong and there were few QTL, but remained moderately high when the number of QTL was >60 (Fig. 5, Supplementary Fig. 6).

**Fig. 6.**
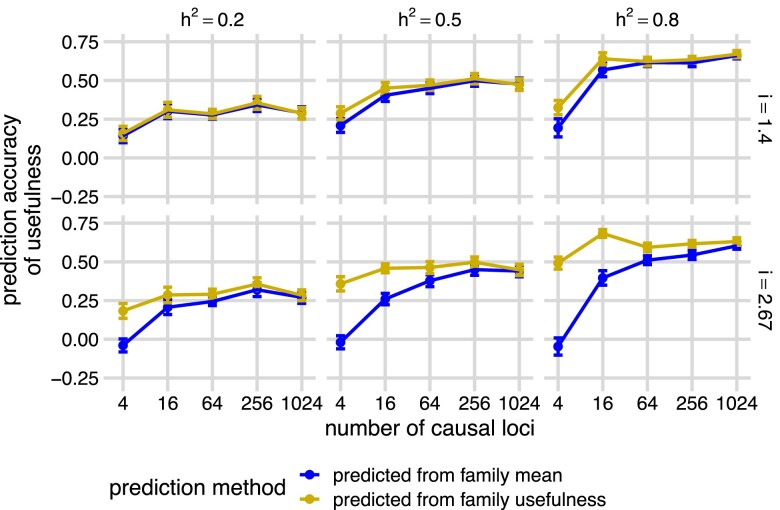
Prediction accuracies of the usefulness across the elite families using a BayesC genomic prediction model. Panels show means and standard error bars of Pearson’s correlations (prediction accuracy, *y*-axis) between the true family usefulness and predicted family mean (blue “predicted from family mean”) or predicted family usefulness (gold “predicted from family usefulness”) based on a BayesC genomic prediction model trained on parental phenotypes simulated with different heritabilities ($h^{2}$) and different numbers of causal loci (*x*-axis), across different selection intensity (*i*), from the families with the highest predicted breeding value individuals as parents. Twenty simulations were run at each combination of $h^{2}$ and number of causal loci. Points and bars show the means and standard errors of accuracies across each *i*. Curves are created by connecting the points of means and show the trend of prediction accuracies across simulations.

Using our previously trained BayesC models, prediction accuracies of usefulness were moderately higher using predicted usefulness for each candidate family than using predicted family means in the high heritability/few QTL/low selection intensity scenarios, so the benefit increased when the selection intensity was high and the number of QTL was small (Fig. 6).

These results suggest that a two-stage crossing design strategy, in which parents are first selected by predicted breeding value and then crosses among these selected parents are designed in a second state, might be an effective method for leveraging usefulness to increase genetic gain, at least when heritability and/or selection intensity is high and the genetic architecture has a small number of QTL. However, the prediction accuracies and selection intensities shown in Fig. 6 are not direct parameters of the traditional breeder’s equation, usually written as $\Delta G=ir\sigma_{A}$. The parameter *i* from the breeder’s equation represents the selection intensity relative to all possible offspring, while the *i* in the usefulness equation is the selection intensity within each family. The parameter *r* from the breeder’s equation represents the accuracy of predicted genetic values of each offspring, while the *r* from Fig. 6 represents the accuracy of predicted average genetic values of the selected offspring in each cross. Additionally, Fig. 6 does not show the population additive genetic variance $\sigma_{A}$, and the second stage of the two-stage crossing design will have less genetic variance because only crosses among the previously selected parents are considered. Therefore, Fig. 6 provides an incomplete picture of the potential benefit of predicting usefulness for increasing genetic gain.

To assess whether the increases in accuracy from predicted usefulness shown in Fig. 6 actually result in meaningful increases in genetic gain, we estimated the genetic gain expected for this two-stage approach and compared it with the expected genetic gain from the one-stage approach that selected crosses from all possible crosses using either predicted usefulness or predicted mean breeding values. The expected genetic gain is the mean genetic value of the top offspring from each of the selected crosses, using a selection intensity of *i* within each cross. For each approach, we created 10 crosses using 20 unique parents. For the two-stage approach, we selected the 40 parents by predicted breeding values, and predicted usefulness values for all 780 candidate crosses, and then chose 10 crosses using a greedy search algorithm, first choosing the cross with the highest predicted usefulness, then adding the next-best cross that did not use any previously selected parents, and so-on until 10 crosses were selected. For the one-stage approach, we predicted family means and family usefulness for all 506,521 possible crosses, and then applied the same algorithm to select 10 crosses using 20 unique parents. Note that a two-stage approach using predicted family means as the cross-section criteria in the second stage would choose identical crosses as the one-stage approach using predicted family means. Since simulating crosses for 506,521 crosses would be prohibitively computationally expensive, we instead simulated populations of gametes from each parental genotype, and predicted family means as the sum of the mean predicted gamete genetic values of each parent, and predicted family variances as the sum of the variances of predicted gamete genetic values.

**Fig. 7.**
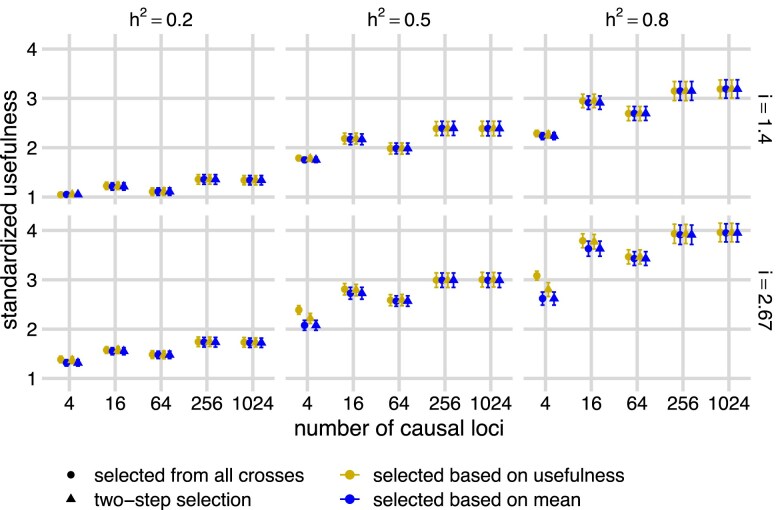
Selection on predicted family usefulness from all possible crosses gave crosses with the highest breeding value usefulness. Panels show means and standard error bars of standardized family breeding value usefulness (*y*-axis) against the number of QTL (*x*-axis) from crosses based on four selection methods across different heritability ($h^{2}$) and selection intensity (*i*). The selection methods included selecting the top 10 crosses among all possible crosses with the highest predicted family usefulness (gold “selected based on usefulness”, circle) without replacement, selecting the top 10 crosses among all possible crosses with the highest predicted family mean (blue “selected based on mean”, circle) without replacement, selecting top 40 ranked parents based on BayesC model predicted breeding values and then selecting the top 10 crosses among them with the highest predicted family usefulness (gold “selected based on usefulness”, triangle) without replacement, and selecting top 40 ranked parents based on BayesC model predicted breeding values and then selecting the top 10 crosses among them with the highest predicted family mean (blue “selected based on mean”, triangle) without replacement.

Figure 7 shows the expected genetic gains in units of $\sigma_{A}$ for each selection strategy. Consistent with the results of Fig. 3, predicting usefulness can increase genetic gain, and never results in lower rates of gain on average. However, except in cases of extreme within family selection intensity, high heritability, and few QTL, the gain is rarely large. In contrast to the impression given by Fig. 6 and to the hypothesis by Zhong and Jannink (2007), there was limited benefit of selecting crosses by predicted usefulness after selecting parents, even when the accuracy of predicted usefulness was high. The two-stage approach never had higher expected genetic gains than the one-stage approach using predicted usefulness.

### Mate selection based on family usefulness provides little benefit in most cases and minimal benefit in some cases

Together, our results show that the true family mean is highly correlated with the true family usefulness under most scenarios in an outcross species like strawberries. Our results validated conclusions made by Zhong and Jannink (2007), in which they stated limited benefit of usefulness in inbred species. We show that the accuracy of computational phasing is unlikely to be the limiting factor in the utility of usefulness predictions for improving genetic gain. Even when phasing is successful, as it appears to be in our strawberry population, and when predicted usefulness values are relatively accurate, predicting usefulness values for potential crosses only has meaningful benefit compared with only predicting family means when the genetic architecture is dominated by few QTL, when the heritability is moderate or high, and when family sizes are so large that very high within family selection intensities can be achieved (Fig. 7, panels $h^{2}=0.5$, $h^{2}=0.8$, and i=2.67 at four QTL). One possible explanation for our high phasing accuracy is that our reference strawberry breeding program includes many parent–offspring pairs which are highly informative for inferring phasing among markers. In other programs without such a strong pedigree structure, lower phasing accuracy may further limit the benefit of usefulness.

One limitation of our study is that we only simulated additive genetic architectures which is justified if we are only interested in population improvement. Under purely additive architectures, the usefulness of a cross is simply a function of the average usefulness of each parent. In this case, usefulness can be used equally as a line or mate selection metric. However, for variety release in a clonally propagated crop like strawberry, the usefulness criterion can be extended to total genetic values where dominance and epistasis effects will additionally be important (Wolfe *et al.* 2021). In this case, if the within family variance in total genetic values becomes large enough so that the variance of $i\sigma_{j}$ is of similar magnitudes to $\mu_{j}$, the importance of usefulness would increase. However, predicting dominance and epistatic effects from genome-wide marker data remains very challenging and generally requires much larger sample sizes than are currently possible in most breeding programs. Wolfe *et al.* (2021) found little benefit from predicting dominance variance. In future work, we could extend our simulations to include dominance and epistatic variance to confirm these intuitions.

### Conclusion

In this study, we discuss whether selecting crosses based on predicted values of the usefulness of each cross could improve the rate of gain in breeding programs. Predicting the usefulness requires predicting the standard deviations of breeding values among the offspring produced by each cross. Our results show that predicting usefulness improves outcomes relative to simply predicting the mean breeding value of each family only in very specific scenarios with high heritability, high selection intensity, and a small number of QTL (Figs. 3 and 4a). However, even in these scenarios, the relative gain in accuracy was small. We have also shown that the true usefulness of the top crosses selected either with predicted family mean or with usefulness only meaningfully differ in those same scenarios (Fig. 7). Due to the rare instances where prediction of usefulness is beneficial, usefulness is rarely useful.

## Supplementary Material

jkae296_Supplementary_Data

## Data Availability

The data used in this study are available at: https://gsajournals.figshare.com/articles/dataset/Supplemental_Material_for_Wang_Feldmann_and_Runcie_2024/26957599. The Supplementary figures are provided. The codes are available at https://github.com/Faye-Wong-stat/family_variance.git. Supplemental material available at G3 online.
