# Supplementary figures and images for: Why usefulness is rarely useful
Source: G3 (Bethesda). 2024 Dec 24;15(3):jkae296. doi: 10.1093/g3journal/jkae296 (PMC11917469; doi:10.1093/g3journal/jkae296)

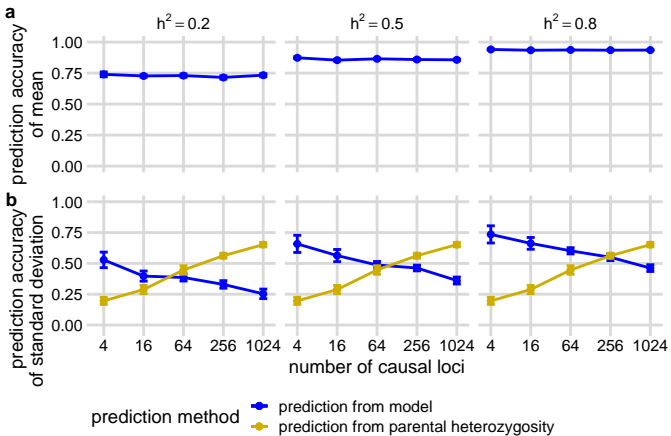

Supplement: jkae296_Supplementary_Data [file jkae296_supplementary_data.zip › Figure_S1_G3-2024-405382.pdf]

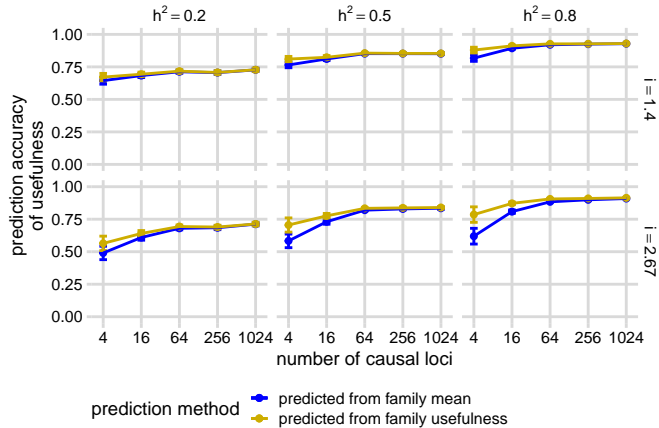

Supplement: jkae296_Supplementary_Data [file jkae296_supplementary_data.zip › Figure_S2_G3-2024-405382.pdf]

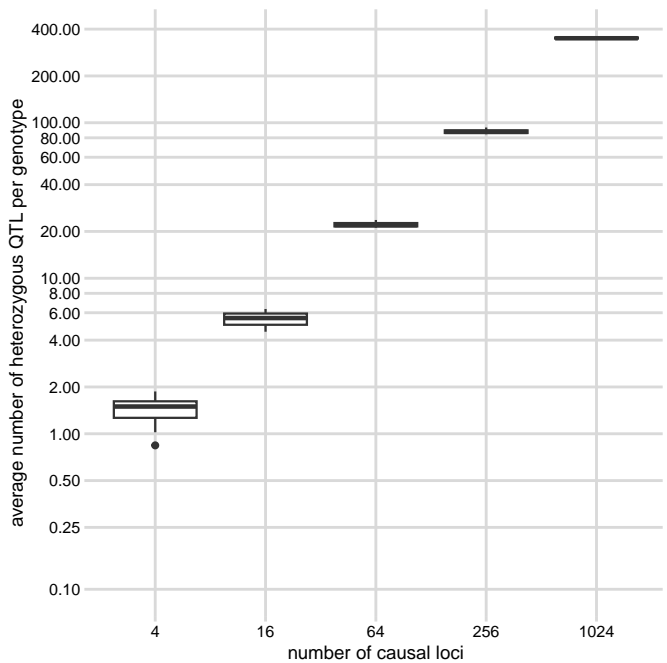

Supplement: jkae296_Supplementary_Data [file jkae296_supplementary_data.zip › Figure_S3_G3-2024-405382.pdf]

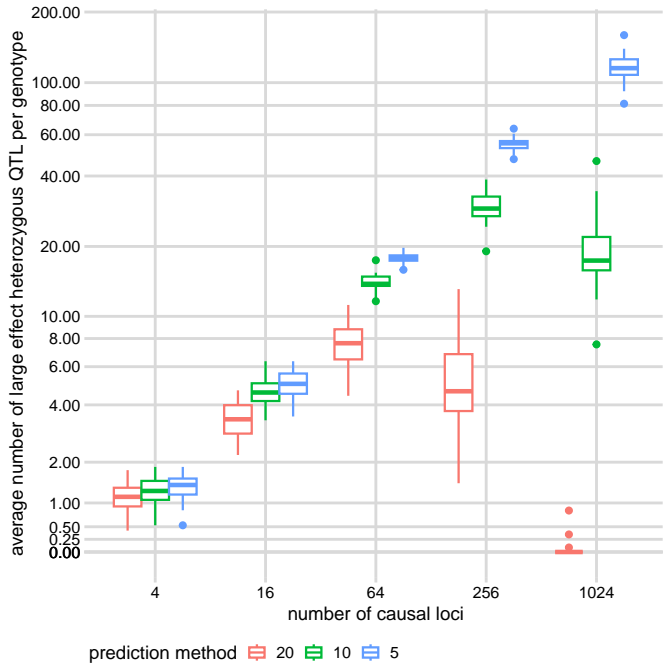

Supplement: jkae296_Supplementary_Data [file jkae296_supplementary_data.zip › Figure_S4_G3-2024-405382.pdf]

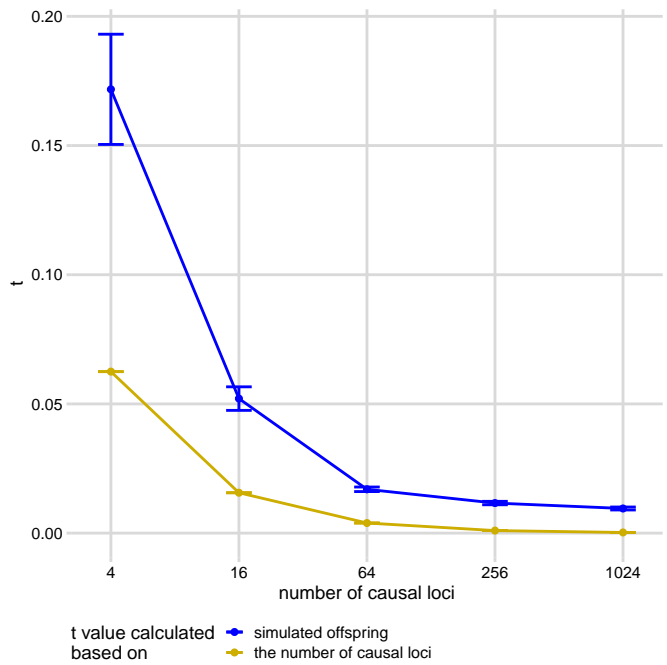

Supplement: jkae296_Supplementary_Data [file jkae296_supplementary_data.zip › Figure_S5_G3-2024-405382.pdf]

correlation between BV mean  
and usefulness

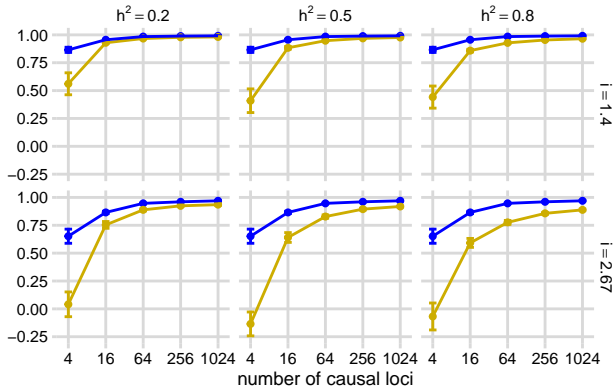

parent type — elite parents — random parents

Supplement: jkae296_Supplementary_Data [file jkae296_supplementary_data.zip › Figure_S6_G3-2024-405382.pdf]

**a**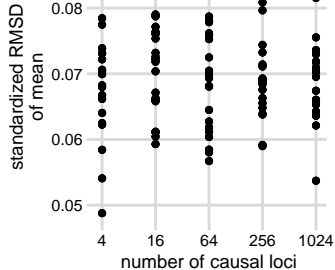**b**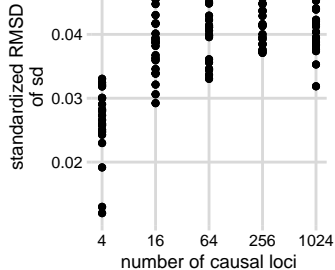

Supplement: jkae296_Supplementary_Data [file jkae296_supplementary_data.zip › Figure_S7_G3-2024-405382.pdf]
